# Bicalutamide Exhibits Potential to Damage Kidney via Destroying Complex I and Affecting Mitochondrial Dynamics

**DOI:** 10.3390/jcm11010135

**Published:** 2021-12-27

**Authors:** Kuan-Chou Chen, Chang-Rong Chen, Chang-Yu Chen, Chiung-Chi Peng, Robert Y. Peng

**Affiliations:** 1 Graduate Institute of Clinical Medicine, School of Medicine, College of Medicine, Taipei Medical University, 250 Wu-Xing St., Sin-Yi District, Taipei 11031, Taiwan; kuanchou@tmu.edu.tw; 2 Department of Urology, Taipei Medical University Shuang-Ho Hospital, New Taipei City 23561, Taiwan; 3 TMU-Research Center of Urology and Kidney, Taipei Medical University, Taipei 11031, Taiwan; 4 International Medical Doctor Program, Vita-Salute San Raffaele University, 20132 Milan, Italy; cherylcherylchen@gmail.com; 5 Program of Biomedical Sciences, College of Arts and Sciences, California Baptist University, Riverside, CA 92504, USA; eugenechen0529@gmail.com; 6 Department of Biotechnology, College of Medical and Health Care, Hungkuang University, No. 1018, Sec. 6, Taiwan Boulevard, Shalu District, Taichung 43302, Taiwan; robertpeng120@gmail.com

**Keywords:** bicalutamide, complex I NDUFB8, NADPH oxidase 4 (Nox4), sirtuins (SIRTs)1/3, PGC1α, glutathione (GSH), superoxide dismutase 2 (SOD2), mitofusins 1/2 (MFN 1/2), optic atrophy 1 (OPA1)

## Abstract

Bicalutamide (Bic) is an androgen deprivation therapy (ADT) for treating prostate cancer, while ADT is potentially associated with acute kidney injury. Previously, we recognized Bic induced renal mitochondria dysfunction in vitro and in vivo via the ROS -HIF1α pathway. Whether OXPHOS complex, as well as mitochondrial dynamics, can be influenced by Bic via modulation of peroxisome proliferator-activated receptor coactivator 1α (PGC1α), NADPH oxidase 4 (Nox4), mitofusins 1/2 (MFN 1/2), optic atrophy 1 (OPA1), and sirtuins (SIRTs) has not been documented. Renal mesangial cell line was treated with Bic (30~60 μM) for the indicated time. SIRTs, complex I, mitochondrial dynamics- and oxidative stress-related proteins were analyzed. Bic dose-dependently reduced mitochondrial potential, but dose- and time-dependently suppressed translocase of the outer mitochondrial membrane member 20 (Tomm 20), complex I activity. Nox4 and glutathione lead to decreased NAD^+^/NADH ratio, with upregulated superoxide dismutase 2. SIRT1 was initially stimulated and then suppressed, while SIRT3 was time- and dose-dependently downregulated. PGC1α, MFN2, and OPA1 were all upregulated, with MFN1 and pro-fission dynamin-related protein I downregulated. Bic exhibits potential to damage mitochondria via destroying complex I, complex I activity, and mitochondrial dynamics. Long-term treatment with Bic should be carefully followed up.

## 1. Introduction

To perform a diversity of physiological processes, kidneys are constructed to be extremely heterogenous, both morphologically and functionally. Podocytes in glomeruli are essential to blood filtration [1], and the proximal tubule is required for reabsorption of filtrate, while the primary function of mesangial cells is to remove trapped residues and aggregated proteins from the basement membrane, thus keeping the filter free of debris [2]. Obviously, to maintain these physiological functions requires a huge amount of energy [3].

Complex I of the mitochondrial oxidative phosphorylation (OXPHOS) system carries out two key activities: the transfer of electrons from mitochondrial matrix NADH to coenzyme Q (CoQ), and the pumping of protons across the inner mitochondrial membrane (IMM), creating a ΔpH_m_ potential across IMM and translocating through F_0_/F_1_ ATPase (ATP synthase) to generate ATP [4,5]. Breakdown or mutations in structural subunits or assembly factors in complex I results in broad biochemical defects, including NAD^+^/NADH ratio imbalance, impaired maintenance of complex I activity and the mitochondrial membrane potential (MMP) [5,6]. Noteworthy is that, molecular pathologically, reduced complex I activity has also been implicated as a contributor to Parkinson’s disease [7], or to the aggravation of folic acid-induced acute renal injury [8]. One potential strategy for treating complex I deficiency is to rescue OXPHOS activity by engaging complex I-independent pathways of entry, often referred to as “complex I bypass” [9].

Mitochondria and NADPH oxidase 4 (NOX4) are the two major sources of reactive oxygen species [10] in kidneys [4,11]. Superoxide dismutase 2 (SOD2) is embedded within the supercomplex I:III:IV to stabilize or locally protect it from ROS damages. Many proinflammatory cytokines, growth factors, and redox active agents can induce SOD2 expression [12,13]. Decrease of SOD2 results in accelerated renal cellular senescence, enhancing tubular damage and glomerular sclerosis upon aging [14]. NOX4 is the most abundant NADPH oxidase isoform in kidneys, which catalyzes the production of hydrogen peroxide (H_2_O_2_) and regulates a diversity of physiological functions [15]. Under physiological conditions, NOX4 proteins are undetectable in the renal mitochondria [16]. NOX4 was suggested specifically to inhibit the activity of electron transport chain (ETC) complex I and decrease its subunit concentration [17]. NOX4 contributes to redox processes involved in diabetic nephropathy, acute kidney injury (AKI), and other renal diseases by activating multiple signaling pathways [18]. Diabetes upregulated NOX4 expression in podocytes and mesangial cells, which was shown to damage glomeruli leading to podocyte loss, mesangial cell hypertrophy, and matrix accumulation [19].

Mitochondrial dynamics, mitophagy, and biogenesis contribute to the supply of healthy mitochondria within cells [20]. The transcriptional coactivator, peroxisome proliferator-activated receptor (PPAR) coactivator 1α (PGC1α) family, is considered as the master regulator network of mitochondrial biogenesis and respiratory function [21,22], which is modulated by extracellular signals controlling metabolism, differentiation, and in some cases, by post-translational modification with energy sensors, such as AMPK and sirtuin 1 (SIRT1) [21]. Attractively, invasive cancer cells use PGC1α to enhance oxidative phosphorylation, mitochondrial biogenesis, and the oxygen consumption rate [23]. Sirtuins are a family of nicotinamide adenosine dinucleotide (NAD^+^)-dependent deacetylases, which regulate cellular proliferation and differentiation, metabolism, response to stress [24]. SIRT1 is required for androgen antagonist-mediated transcriptional repression and growth suppression [25], the maintenance of glomerular barrier function, antifibrosis effects, antioxidative stress effects, and regulation of mitochondria function and energy metabolism [26]. SIRT3 regulates numerous aspects of mitochondrial function, including the control of β-oxidation, the ETC, ATP production, the urea cycle, ROS detoxification, and mitochondrial dynamics [24,27].

Bicalutamide (Bic) is frequently used in androgen deprivation therapy [1] for treating prostate cancer. ADT-induced hypogonadism exhibits the potential leading to AKI [28]. Bic frequently shows adverse effects associated with cardiovascular and renal damages [28,29], as well as hepatotoxicity [30]. Previously, we recognized Bic induced renal damages in vitro and in vivo via the ROS-HIF1α pathway [31]. We hypothesize that Bic may further damage mitochondrial structure and function via injuring the OXPHOS complex, mitochondrial dynamics. To uncover this, we carried out this study.

## 2. Materials and Methods

### 2.1. Cell Culture

The rat mesangial cell (RMC cell line), derived from the kidney tissue of the 3-month-old male rat belonging to the Sprague-Dawley strain, was purchased from the Bioresource Collection and Research Center (BCRC, Hsin-Chu, Taiwan). RMCs were cultured in modified 85% DMEM containing 25 mM glucose, 15% FBS, 4 mM L-glutamine, 1.5 g/L of sodium bicarbonate, 0.4 mg/mL of G418, and 0.4% phosphate-buffered saline (PBS) and incubated at 37 °C under a 5% CO_2_ atmosphere. After cells completely adhered, the medium was replaced with 2% charcoal FBS DMEM and incubated overnight. Different cell density was seeded for each following experiment. The experimental groups consisted of the control, 30 and 60 μM of Bic in DMEM with 2% charcoal FBS, respectively. As reference, the results of cell viability affected by Bic in a time- and dose-dependent manner previously reported by us is shown in Figure S1 [32]. As usual, the cells were cultured for 1~48 h and used for performing following examinations at 24 and 48 h, respectively. Worth mentioning is that, previously, we found ROS appeared very early at 1 h [31]; hence, for an efficient tracking of the related oxidative parameters, exceptionally, the appearance of NOX4 was examined early at 1 and 24 h, and that of NAD^+^ was detected at 3 h, while glutathione (GSH) measurement immediately followed early, at 6 h.

### 2.2. Measurement of Mitochondrial Membrane Potential (MMP)

RMC cells with a density 4 × 10^4^/mL was prepared with 2% charcoal FBS medium and seeded onto µ-Slide 4 Well Chamber Slide (Cat. No:80426, ibidi GmbH, Gräfelfing, Germany) and incubated for 24 h at 37 °C under 5% CO_2_ atmosphere until adhered. The medium was removed and replaced with 2% charcoal FBS containing Bic (0, 30, and/or 60 μM, respectively) and incubated for 24 h at 37 °C under 5% CO_2_ atmosphere. After the medium was removed, to each well, 150 nM MitoView^TM^633 (#70055-T, Biotium, Inc., Fremont, CA, USA) and 20 μM Hoechst^®^ 33342 (#62249, Thermo Fisher Scientific, Waltham, MA, USA) (Hoechst stock solution 1:2000 in PBS) were simultaneously added at a sufficient amount to cover the cells and incubated at 37 °C under 5% CO_2_ atmosphere for 15 min, avoiding direct sunlight. The staining solution was removed, and the cells were rinsed repeatedly with PBS three times. The fluorescence was immediately imaged. The cells with far-red fluorescent mitochondrial dye MitoView™ 633 were measured at λ_excitation_/λ_emission_ = 622/648, whereas Hoechst fluorescence was imaged at wavelength λ_excitation_/λ_emission_ = 350/461 [33].

### 2.3. Western Blotting Analysis

After treatment with Bic, the total- and mitochondrial proteins of RMC cells were extracted at the indicated time with ProteoExtract^®^ Cytosol/Mitochondria Fractionation Kit (Merck Millipore, Burlington, MA, USA).

The protein contents were analyzed by Bradford Protein Assay Kit (Integrated Bio, Taipei, Taiwan) according to the manufacturer’s instruction. The sample proteins were heated at 100 °C for 10 min before loading onto precasted 7.5~15 % SDS-PAGE to carry out separation. Aliquots of samples containing protein 50 µg/µL were electrotransferred onto the PVDF membrane in transfer buffer for 1 h. The nonspecific binding to the membrane was blocked for 1 h at room temperature with 5% nonfat milk in TBS buffer. The membranes were then incubated for 16 h at 4 °C with anti-Tomm 20 (GTX133756), anti-NOX4 (GTX121929) and anti-Mitofusin 1 (MFN1) (GTX133351) (GeneTex, Inc., Irvine, CA, USA), anti-NDUFB8 (tccua 57905), anti-SIRT1 (tcea19648), anti-SIRT3 (tcna3378), anti-PGC1α (tcea3326), anti-Mitofusin 2 (MFN2) (tcea20884) (Taiclone Biotech Co., Taipei, Taiwan), anti-SOD2 (Cell Signaling Technology, Danvers, MA, USA), anti-OPA1 (NBP2-59770, Novus Biologicals, Centennial, CO, USA), and anti-DRP1 (sc-271583, Santa Cruz Biotechnology, Inc., Santa Cruz, CA, USA). Anti-β actin (NB600-501, Novus Biologicals, Centennial, CO, USA) and anti-HSP60 (GTX110089 GeneTex, Inc., Irvine, CA, USA) were used as the internal controls as indicated [34]. The luminescence was detected using ECL Western Blotting Substrate Kit (Waltham, MA, USA), and the signals were visualized using the Luminescent Image Analyzer LAS-4000 (Fujifilm, Tokyo, Japan).

### 2.4. CellTiter-Glo Luminescence-Based Assay for Preliminary Testing the Complex I Function

The RMC cells were seeded onto a 96-well plate at density of 1 × 10^4^/well, cultured in modified 85% DMEM containing 25 mM glucose with 2% charcoal FBS, and then incubated at 37 °C under 5% CO_2_ atmosphere for 24 h until adhered. The plate was rinsed three times with PBS, and the media was replaced with DMEM containing no glucose or FBS. Bic at doses 0, 30, and 60 μM (previously dissolved in 2% charcoal medium containing 10 mM galactose without glucose) was added and incubated at 37 °C under 5% CO_2_ atmosphere for additional 24 h. To each well, 125 nM antimycin and 100 μL ATP reagent were added and incubated at 37 °C for 45–90 min. The ATP content was measured according to the method modified by Vafai (2016) [35] using the Cell Titer Glo Luminescent Cell Viability Assay kit (Promega G7570, Promega Corporation, Madison, WI, USA). The Cell Titer Glo reagent was prepared according to the manufacturer’s protocol and diluted to 33.3% with PBS, 15 μL of which were added to each well and allowed to incubate at room temperature for 10 min, avoiding direct sunlight, to stabilize the spectral stability. The luminescence of each well was measured using a SpectraMax^®^ reader (Molecular Devices, San Jose, CA, USA) and normalized by expressing it as a fold-change relative to the average luminescence of the appropriate negative control wells on the same plate.

### 2.5. Complex I Immunofluorescent Staining

RMC cells at a density 4 × 10^4^/mL prepared with 2% charcoal FBS medium were seeded onto 1 µ-Slide 4 Well Chamber Slide (ibidi 80284, poly-L-lysine coated) and incubated for 24 h at 37 °C under 5% CO_2_ atmosphere until adhered. The medium was removed and replaced with Bic (0, 60 μM)-containing medium prepared from 2% charcoal FBS medium and incubated for additional 24 h. The medium was removed, the cells were rinsed twice with PBS, and the washings were discarded. A paraformaldehyde solution (4%) was added to fix the cells at ambient temperature for 20 min. After antigen retrieval with citrate buffer (pH 6.0) for 15 min, 10% BSA blocking solution was applied and left at ambient temperature for 1 h. Primary antibody Complex 1 (GTX105835, GeneTex, Inc., Irvine, CA, USA) in BSA-PBST was added and kept at 4 °C overnight. The cells were rinsed thrice with PBS, and anti-rabbit secondary antibody in 1% BSA-PBST was added and left to stand at room temperature for one hour. The cells were rinsed thrice with 1% PBS-PBST. The washings were removed, and Hoechst 33342 (Thermo Fisher Scientific, Waltham, MA, USA) in PBST was added, left at ambient temperature for 15 min. The cells were rinsed thrice with PBS and then subjected to fluorescence imaging.

### 2.6. Measurement of Complex I Activity

By following the instruction given by Cayman Chemical (Ann Arbor, MI, USA), MitoCheck^®^ Complex I Activity Assay Kit (Cayman, #700930) was used for the assay of complex I activity. In brief, all the required reagents were mixed to prepare solutions A and B. To each well, 50 μL of solution A was added, then 20 μL of Bic (0, 30, and 60 μM)/well, or 20 μL of rotenone (2 μM)/well as the positive control. Finally, after adding 30 μL of solution B, the optical density was successively read by SpectraMax^®^ reader (Molecular Devices, San Jose, CA, USA) at 340 nm at intervals of 30 s for an entire 15-min period.

### 2.7. Assay for Glutathione

RMC cells at a density 6 × 10^5^/mL in 2% charcoal FBS medium were seeded onto a 10 cm dish and incubated for 24 h at 37 °C under 5% CO_2_ atmosphere until adhered. Bic at 0, 30, and 60 μM prepared with 2% charcoal FBS medium was added and further incubated for 6 h. The medium was removed, and the cells were rinsed twice with PBS. The ice-cooled 1 × MES buffer 1 mL was added, and the cells were harvested by scratching down the cells. The cells were disrupted by ultrasonication and centrifuged at 10,000× *g* at 4 °C for 15 min. The supernatant was transferred into a new tube. The content of glutathione (GSH) was assayed with Glutathione Assay Kit (Cayman Chemical, Ann Arbor, MI, USA).

### 2.8. Assay for NAD^+^/NADH

Following the instruction given by Cayman Chemical (Ann Arbor, MI, USA), 100 μL of RMC cells at a density 2 × 10^5^/mL (previously prepared in 2% charcoal FBS medium) was seeded onto 96-well plate and incubated at 37 °C for 24 h under 5% CO_2_ atmosphere until adhered. The medium was changed to Bic (0, 30, 60 μM)-containing medium prepared from 2% charcoal FBS medium. The sampling time was set at 3, 24, and 48 h. The NAD^+^ content of which was measured with NAD^+^/NADH Cell- Based Assay Kit (Cayman, #600480). The additional early observation at 3 h was carried out with the purpose of checking the time course influence by oxidative stress.

### 2.9. Statistical Analysis

Statistical analyses were performed using SPSS 10.0 computer statistical software (SPSS, Chicago, IL, USA). Analysis of variance (ANOVA) was used with Tukey’s test to analyze variances and the significance of the difference between means of different groups. The statistical significance of the difference was judged by confidence levels of *p* < 0.05, *p* < 0.01, *p* < 0.001.

## 3. Results and Discussion

### 3.1. Membrane Potential Was Reduced by Bicalutamide Therapy

**Figure 1:**
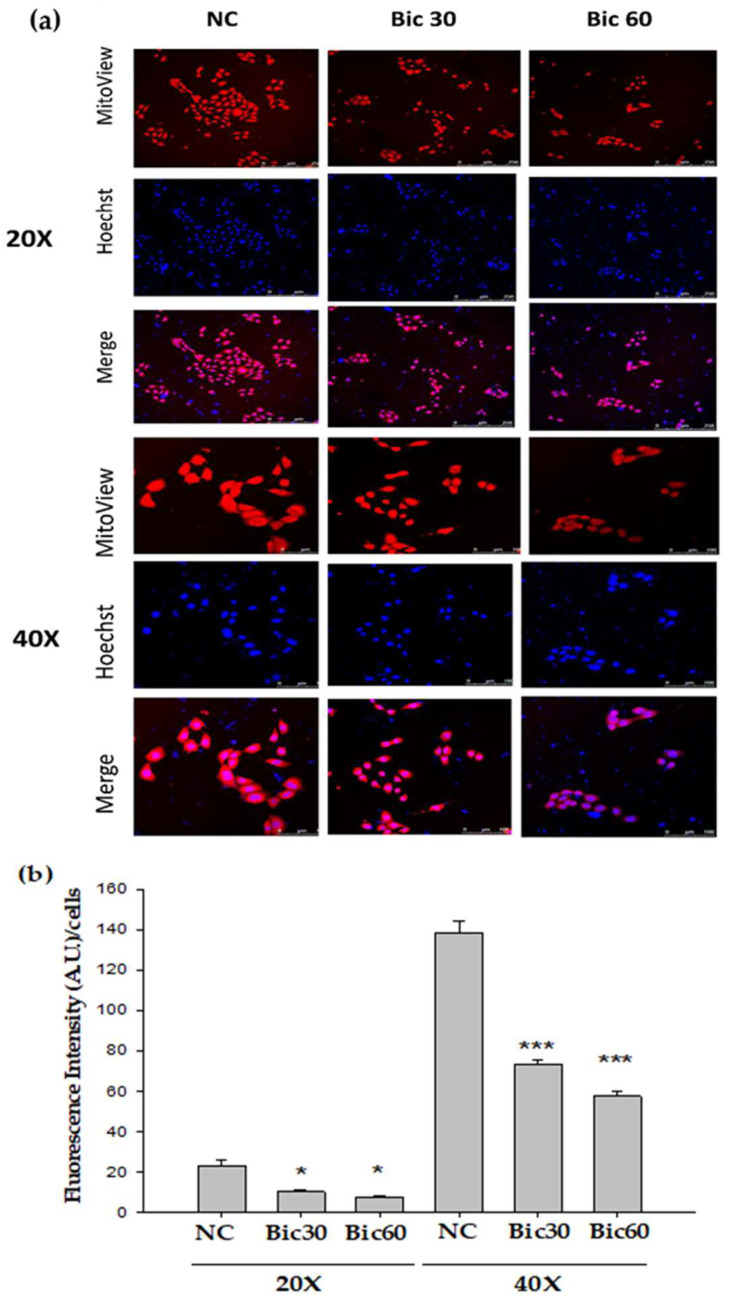
Mitochondrial membrane potential (Δψm) monitored by Mitoview 633^®^ in RMC cells decreased following Bicalutamide treatment. (**a**) RMC cells treated with Bicalutamide after 24 h were incubated with Mitoview 633^®^ (red color) for 15 min, 37 °C. Hoechst 33342 was used to stain nuclei (blue color). (**b**) The quantified bar diagram from images analyzed by fluorescence microscopy. (*n* = 3, NC: normal control, Bic 30, 60: bicalutamide 30 μM and 60 μM. Magnification: 20× for upper three panels, 40× for lower three panels. * *p* < 0.05, *** *p* < 0.001 compared to NC).

The MMP (Δψ_m_) in RMC was reduced by Bic in a dose-dependent manner (Figure 1a). MitoView™ 633 is a far-red fluorescent mitochondrial dye, staining with which is dependent on MMP, and can be used as a key indicator of cell health or injury in intact cells [36]. Recently, we showed Bic damaged kidneys via causing mitochondrial dysfunction elicited by ROS attack induced by HIF-1α [31].

The reductive transfer of electrons through ETC protein complexes I–IV in the inner mitochondria membrane provides the energy to drive protons against their concentration gradient across the inner mitochondrial membrane (out of the mitochondrial cytoplasm) [36]. This results in a net accumulation of proton (H^+^) outside the membrane, which then flows back into the mitochondria through the ATP-generating F1/F0 ATP-synthase (Complex V), thus producing ATP and completing the ETC [36]. The MMP arising from normal function of ETC ranges from −136 to −140 mV, which has been considered optimal for maximum ATP production in all living organisms [37]. By approximation, Bic at 30–60 μM altered the MMP down to below a fluorescence intensity 75 compared to 140 of the control (Figure 1b).

Worth of note is that the decrease of MMP might not be entirely caused by proton leak but, also possibly, by respiratory chain defects [38].

### 3.2. Tomm 20 Was Downregulated by Bicalutamide

**Figure 2:**
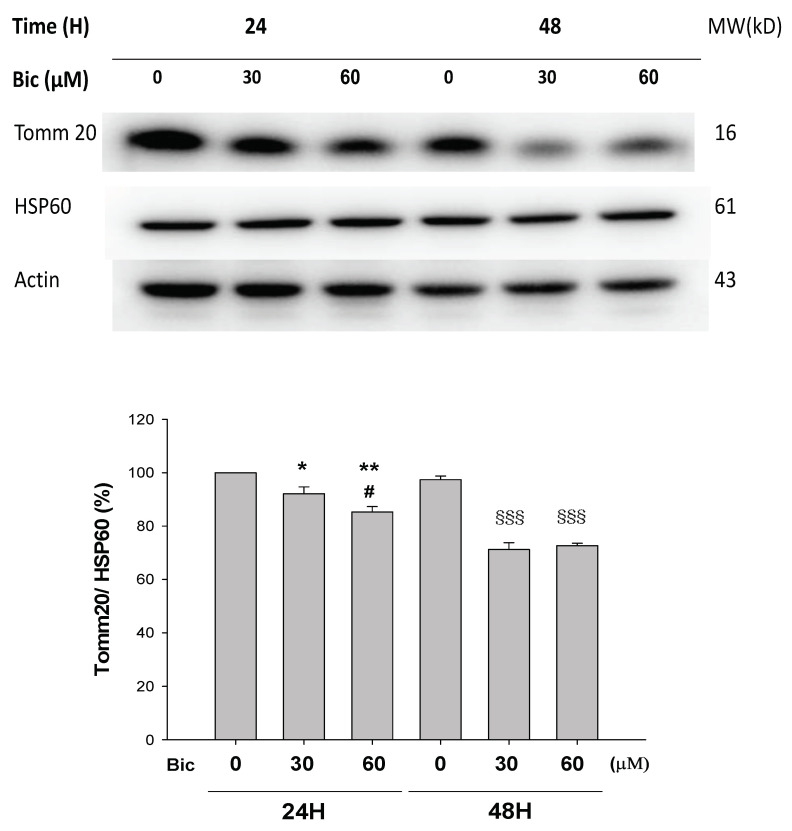
Representative western blot and densitometric analysis showing level of mitochondrial proteins Tomm20 in RMC cells following 0~60 μM of bicalutamide treatment for 24 and 48 h (HSP60 is shown as normalization control). Bar graph represents the mean ± SD of three independent experiments. * *p* < 0.05, ** *p* < 0.01 compared to Bic 0 μM at 24 h. ^#^ *p* < 0.05 compared to Bic 30 μM at 24 h. ^§§§^ *p* < 0.001 compared to Bic 0 μM at 48 h.

Tomm 20 (Translocase of the outer mitochondrial membrane member 20) is a mitochondrial import receptor subunit for transporting cytosolically synthesized mitochondrial preproteins into the mitochondria, which includes the subunits of the OXPHOS machinery [39], cytochrome P450 monooxygenase system (CYP), the major enzymes responsible for metabolizing medications [40,41]. Expression of Tomm 20 protein has been shown to correlate with both mitochondrial mass and respiratory function [42], as well being directly related to OXPHOS when measured by oxygen consumption rates [43]. Tomm 20 was downregulated by Bic challenge in a dose-dependent manner (Figure 2). Mitochondrial damage has been demonstrated as a cause for side effects of many drugs and toxins [44]. The downregulation of Tomm 20 could mean a default of translocating cytochrome P450 monooxygenase and other essential preproteins, which supposedly may enhance Bic toxicity.

Recently, Tomm 20 was shown to be overexpressed in various cancers, directly impacting the mitochondrial function, including ATP production and maintenance of membrane potential [40]. Inhibition of Tomm 20 resulted in significant decreases in cell proliferation, migration, and invasion [40]. Speculatively, Bic inhibition on Tomm 20 may lead to similar outcomes in RMC cells, including decrease in membrane potential (Figure 1).

### 3.3. Decreased ATP Production May Be Related to Complex 1 Defect

As mentioned previously, even slight changes of MMP may result in dramatic decrease in ATP production and intensive increase in ROS production [37].

**Figure 3:**
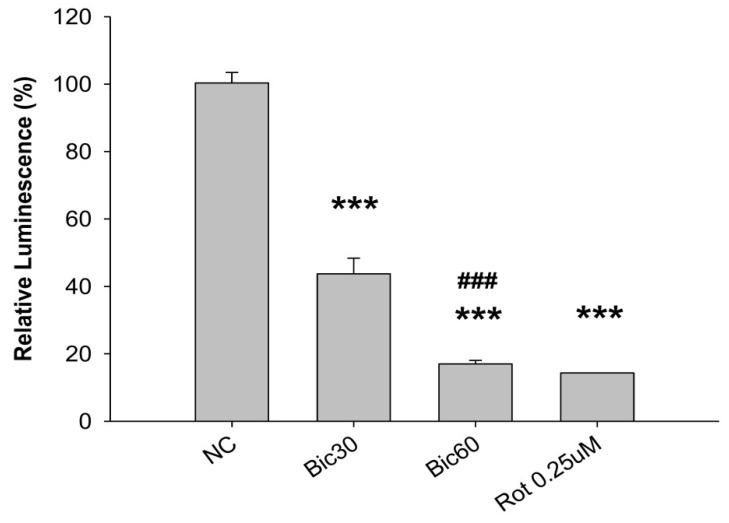
CellTiter-Glo luminescence-based complex I preliminary assay. The ATP content within the RMC cells treated with bicalutamide for 24 h was measured using the CellTiter Glo Luminescent Cell Viability Assay kit (Promega G7570) in the presence of 125 nM antimycin. The luminescence of each well was measured using a Molecular Device SpectraMax^®^ reader and normalized by expressing it as a fold-change relative to the average luminescence of the appropriate negative control wells on the same plate. The 0.25 μM of rotenone (Rot) is shown as positive control for complex I inhibition. Bar graph represents the mean ± SD of three independent experiments. *** *p* < 0.001 compared to normal control (NC). ^###^ *p* < 0.001 compared to Bic 30 μM.

After treatment with Bic at 30 and 60 μM, the relative luminescence, a signal positively correlated with the amount of ATP present, was dose dependently reduced to 43.7 ± 4.7% and 17.0 ± 1.1% comparing with 100 ± 3% and 14.3 ± 0.1% in the negative and the positive rotenone (0.25 μM) controls (Figure 3) under conditions without glucose addition and inhibited complex III. Speculatively Bic could decrease ATP production via damaging complex I (Figure 3), a critical interest of this article. As is well known, complex I is the major entry point for electrons to the respiratory chain, which plays the role as the rate-limiting step in overall respiration and related energy metabolism [4].

### 3.4. Complex 1 Activity Was Downregulated by Bicalutamide Therapy

**Figure 4:**
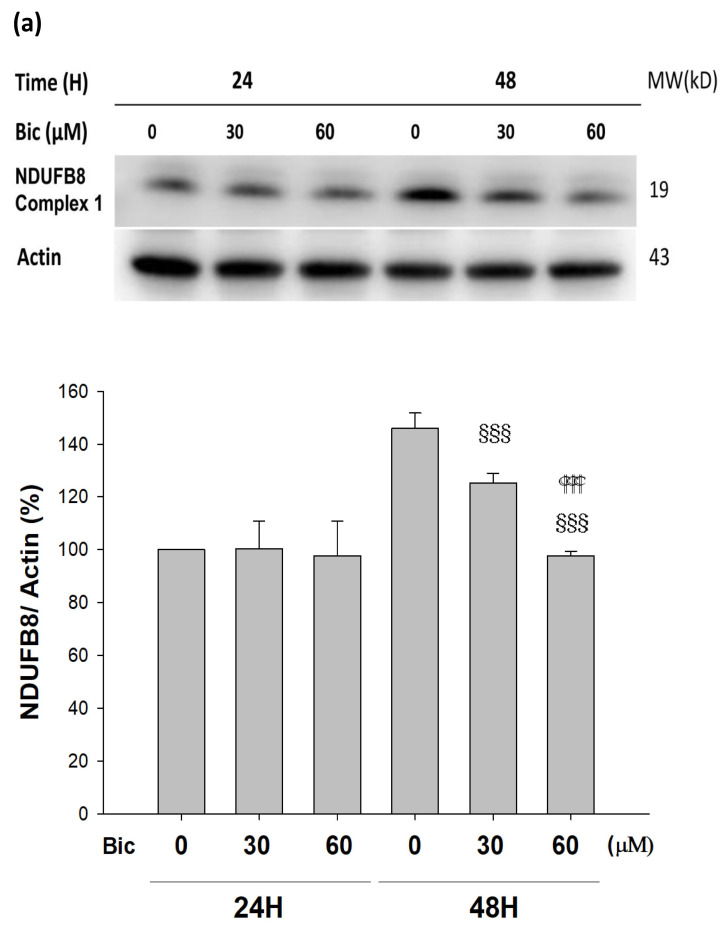

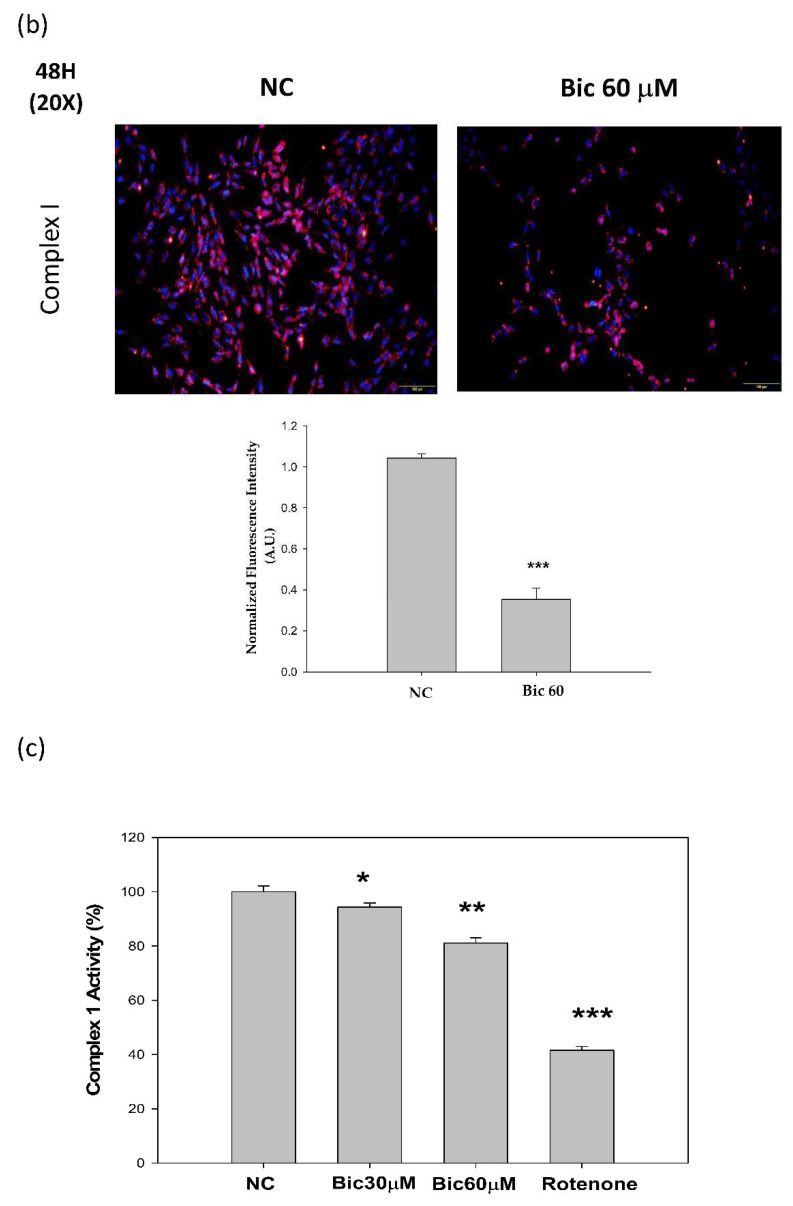
Bicalutamide induces mitochondria respiratory complex I deficiency. Representative western blot and densitometric analysis showing level of NDUFB8 proteins in RMC cells following 0~60 μM of bicalutamide treatment for 24 and 48 h (β-Actin is shown as normalization control). Bar graph represents the mean ± SD of three independent experiments: ^§§§^ *p* < 0.001 compared to Bic 0 μM at 48 h. 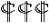 *p* < 0.001 compared to Bic 30 μM at 48 h (**a**). RMC cells were treated with 0~60 μM of bicalutamide for 48 h and subjected to immunofluorescence stain (**b**) and activity assay of complex I at 48 h (**c**). Here, 0.25 μM of rotenone is shown as positive control for complex I inhibition. Bar graph represents the mean ± SD of three independent experiments. * *p* < 0.05, ** *p* < 0.01, *** *p* < 0.001 compared to normal control (NC).

NDUFB8 (NADH:Ubiquinone Oxidoreductase Subunit B8) is one of thirty-eight nuclear-DNA encoded subunits, together with seven mitochondrial-DNA encoded subunits and other assembly proteins, integral to the assembly of complex I [4]. On treating with Bic, it seemed there was no response of NDUFB8 within the first 24 h (Figure 4a); however, significant dose-dependent inhibition on NDUFB8 occurred at 48 h to 86 and 67% respectively, by Bic 30 and 60 μM, compared to control (Figure 4a). Immunofluorescent staining also showed complex I was apparently downregulated by Bic 60 μM at 48 h (Figure 4b). The increase of NDUFB8 in the control and Bic-treated groups at 48 h compared to 24 h may be caused by two reasons, the prolonged exposure to glucose medium [45] and the cell proliferation [32]. Covington and Schnellmann (2012) demonstrated that prolonged exposure to glucose elevated protein expression of NDUFB8 and ATP synthase β [45]. The overall time–activity profile distinctly implicated bioenergetic failure due to inhibition of complex I activity by Bic-ADT therapy in a dose-dependent manner (Figure 4c).

Complex I deficiency is the most common OXPHOS disorder in humans and defects [46]. Inhibition of mitochondrial respiratory chain complex I by rotenone had been found to induce cell death via enhancing the amount of mitochondrial reactive oxygen species production in a variety of cells [47]. In the mice model of folic acid-induced acute kidney injury, inhibition of mitochondrial complex I activity aggravated renal injury, mitochondrial damage, oxidative stress, cell apoptosis, and inflammation [8].

### 3.5. Expression of Nox4 Was Suppressed by Bicalutamide Therapy

**Figure 5:**
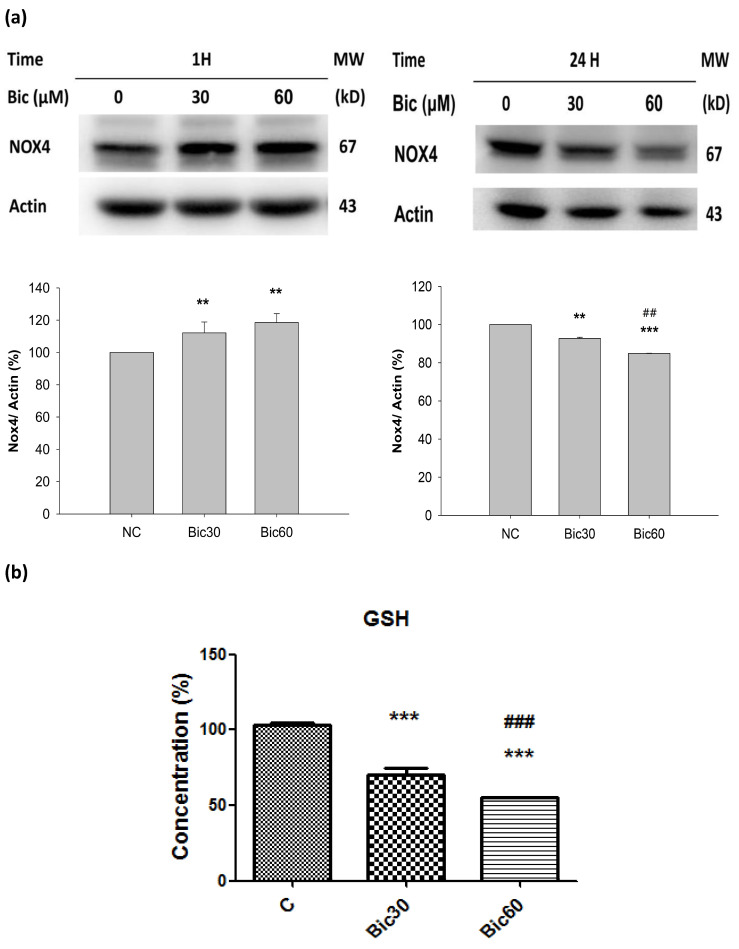
(**a**) Representative western blot and densitometric analysis showing level of NOX4 protein in RMC cells following 0~60 μM of bicalutamide treatment for 1 and 24 h (β-Actin is shown as normalization control). (**b**) The normalized intracellular glutathione (GSH) level in RMC cells treated with 0, 30, and 60 μM of bicalutamide for 6 h. Bar graph represents the mean ± SD of triplicate experiments. ** *p* < 0.05, *** *p* < 0.001 compared to normal control (**c**). ^##^ *p* < 0.05, ^###^ *p* < 0.001 compared to Bic 30 μM.

The level of Nox4 initially dose-dependently increased in the first hour and virtually declined at 24 h after treatment with Bic (Figure 5a).

Nox4 was reported to bind to mitochondrial complex I proteins, but, under basal physiological conditions in cardiac and renal mitochondria, its expression is below the detection limits and does not contribute to ROS formation [16]. The complex I-associated Nox4 can be activated only at reduced oxygen tension. Nox4 has an unusually high K_m_ for oxygen (~18%) [48]. The kinetic mechanism of H_2_O_2_ formation by Nox4 is as a function of oxygen concentration throughout a physiological range of pO_2_ values which responds rapidly to changes in pO_2_, allowing it to function as an oxygen sensor [48].

Nox4 is an oddity among members of the Nox family of NADPH oxidases; around %P_O2_ = 18%, Nox4 generate ROS in proportion 90% H_2_O_2_ with 10% superoxide anions, and that it is constitutively active [48].

Literature indicated that, normally, the hypoxic induction of HO-1 requires an oxygen tension (P_O2_) below 0.5% O_2_ [49], while, in our experiment elsewhere, Bic at 60 μM did not induce any HO-1 (not shown), implicating Bic created a microenvironment with P_O2_ = 0.5~1%; literally, such a state was named “a pseudohypoxia state” by Williamson et al. [50].

Nox4, localized in complex I [16], catalyzes the transfer of electron from NADPH to reduce oxygen. Acting as an oxidative sensor, Nox4 was expressed early at the first hour after Bic treatment (Figure 5a). During this period, the production of ROS in form of H_2_O_2_ was extensively proceeded [31], which, in turn, destroyed GSH (Figure 5b) and subsequently reduced the content of NADPH [51]. As found, the cytosolic glutathione level was dose-dependently reduced to 70.6 ± 2.9% and 55.1 ± 1.1% by Bic at 30 and 60 μM (Figure 5b). As a consequence, Nox4 expression at 24 h was inhibited (Figure 5a), and the Nox4-originated ROS production would be also terminated at the same time.

Alternately, Bic plays the role as a co-inhibitor on histone deacetylase (HDAC), as well as AR antagonists. Inhibition of HDAC reduced Nox4 expression (Figure 5a) and H_2_O_2_ generation [10,52].

### 3.6. Level of NAD^+^ Was Severely Suppressed by Bicalutamide Therapy

**Figure 6:**
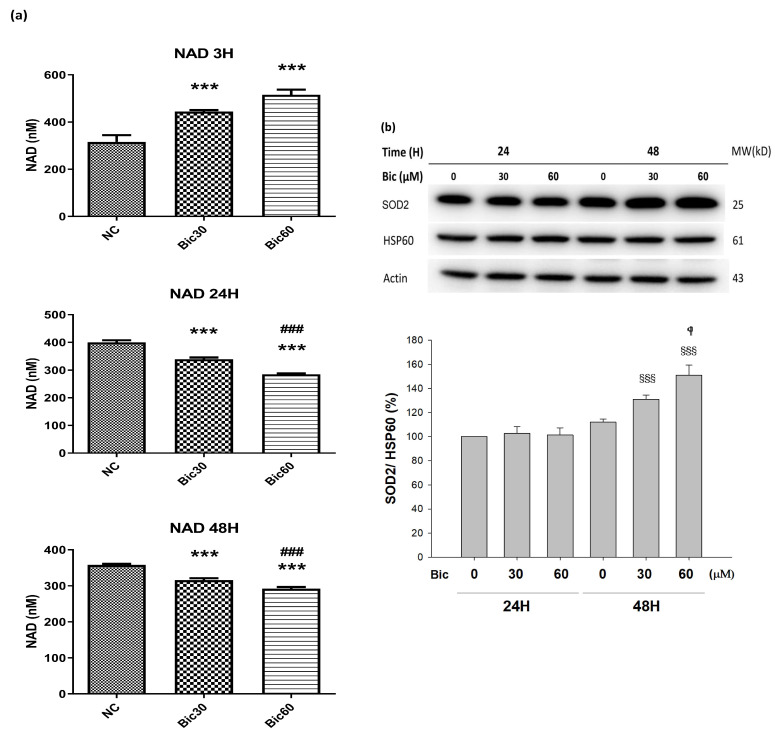
(**a**) Intracellular NAD^+^ level in RMC cells treated with 0, 30, and 60 μM of bicalutamide at 3, 24, and 48 h. (**b**) Representative western blot and densitometric analysis showing level of SOD2 protein in RMC cells following 0~60 μM of bicalutamide treatment for 24 and 48 h (HSP60 is shown as normalization control). Triplicate experiments were performed and expressed as mean ± SD. *** *p* < 0.001 compared to the normal control. ^###^ *p* < 0.001 compared to Bic 30 μM. 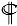 *p* < 0.05, ^§§§^ *p* < 0.001 compared to Bic 30 μM at 48 h.

After treated with Bic, the level of NAD^+^ was seen to increase from 315 μM in control to 444 and 530 μM at Bic 30 and 60μM individually until 3 h, and then declined from 400 μM in control to 340 and 285 μM for Bic 30 and 60 μM at 24 h (Figure 6a). The level of NAD^+^ was continued to decrease from 358 μM in control to 316 and 282 μM for Bic 30 and 60 μM at 48 h (Figure 6a).

Yu demonstrated that elimination of NOX4 activity promoted reductive stress and sensitized the heart to ischemic injury [53]. Overexpression of NOX4 of the hearts exhibited the increase in NAD^+^/NADH and cause significant oxidative stress [53]. Moreover, for well coupled rat liver mitochondria, as the oxygen concentration was lowered, increased cytochrome *c* reduction was observed to begin at oxygen concentrations slightly greater than 20 μM (%P_O2_ = 1.48%) [54], which may be associated with the aforementioned phenomenon.

Complex I catalyzes the first step of NADH oxidation and then elevates the NAD^+^/NADH ratio. Literature indicated that complex I was inactivated by NADPH oxidase NOX4 [17]. Complex I deficiency led to declines in NAD^+^ levels and NAD^+^/NADH redox imbalance, which can overload mitochondrial ETC [50,55].

Previously, we reported that treatment with Bic at 30 and 60 μM for 1 h stimulated huge production of ROS [31], and, herein, we showed that NOX4 was elevated at 1 h (Figure 5a), which might consequently stimulate the over production of NAD^+^ at 3 h (Figure 6a), immediately following the overexpression of NOX4 (Figure 5a); however, both NOX4 and NAD^+^ then declined after 24 h (Figure 5a, Figure 6a). As a result, complex I deficiency was revealed at 48 h (Figure 4c).

In AKI, substantial decreases in the levels of NAD^+^ may impair energy generation. Ultimately, it would damage the function of selective solute transport in kidney, attenuating long-term profibrotic responses and leading to chronic kidney disease [56]. Worth noting is that renal mesangial cell hypertrophy is also characterized by reduced NAD^+^ content [57].

### 3.7. SOD2 Was Upregulated by Bicalutamide Therapy

The manganese superoxide dismutase (MnSOD, SOD2) level was dose-dependently upregulated to 130.9 ± 3.7 and 151.0 ± 8.4%, respectively, after treating with Bic 30 and 60 μM for 48 h (Figure 6b).

SOD2 is a key antioxidant enzyme associated with complex I of mitochondria in eukaryotes [58]. SOD2 defenses against ROS in living cells via catalyzing the redox disproportionation of ^●^O_2_^−^ produced from complex I into H_2_O_2_ and molecular oxygen [12,59]. H_2_O_2_, in turn, is reduced to water by catalase, glutathione peroxidases (GPx), and peroxiredoxins (Prx) on the expense of glutathione (GSH by GPX, glutathione peroxidase) or reduced thioredoxin (TRX_R_ by PRX, peroxiredoxin) [58]. Suggestively, Bic stimulated production of huge amount of superoxide anions (^●^O_2_^−^) [31], resulting in depletion of glutathione (Figure 5b). Excessive superoxide anions (^●^O_2_^−^) play important roles in the pathogenesis of mitochondrial dysfunction associated cardiovascular and renal diseases [58,60].

On the other hand, the complex I deficiency raised electron leakage to significantly initiate intensive production of ROS in form of superoxide anion, which, in turn, induced high level of SOD2 (Figure 6b).

### 3.8. SIRTs Were Downregulated by Bicalutamide Treatment

Both SIRT1 and SIRT3, commonly using NAD^+^ as their coenzyme, are highly associated with energy production and cell viability protection [24].

**Figure 7:**
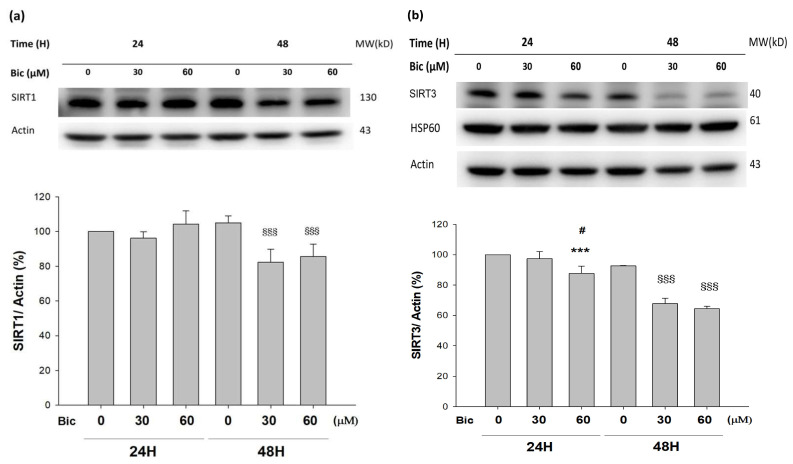
Representative western blot and densitometric analysis showing level of SIRT1 (**a**) and SIRT3 (**b**) proteins in RMC cells following 0, 30, and 60 μM of bicalutamide treatment for 24 and 48 h (β-Actin was used as normalization control). Bar graph represents the mean ± SD of triplicate experiments: *** *p* < 0.001 compared to Bic 0 μM at 24 h. ^#^ *p* < 0.05 compared to Bic 30 μM at 24 h. ^§§§^ *p* < 0.001 compared to Bic 0 μM at 48 h.

SIRT1 was downregulated at 48 h post treatment of Bic (Figure 7a), while SIRT3 was downregulated starting from 24 h in a time- and dose-dependent manner (Figure 7b), which lent support to the relevant role of SIRT3 in association with functions of complex I in mitochondria.

SIRT3, acting as the main mitochondrial deacetylase, critically regulates cellular ROS production and detoxification [61]. SIRT3 prevented p53-induced mitochondrial dysfunction and neuronal damage in a deacetylase activity-dependent manner [62]. In SIRT3 deficient embryonic fibroblasts, SIRT3 interacts with the 39 kDa protein NDUFA9, a subunit of complex I in the mitochondrial electron transport system, thereby activating complex I and increasing ATP production [63].

On the other hand, SIRT1, serving as the key molecule in glucose, lipid, and energy metabolism [26], deacetylates target proteins using the coenzyme NAD^+^ and is, therefore, linked to cellular energy metabolism and the redox state through multiple signaling and survival pathways [24,64]. SIRT1 exhibits renal protective effects and maintains glomerular barrier function via deacetylating p53 and upregulating catalase to reveal multiple activities, including anti-apoptotic, anti-oxidative, anti-inflammation, and anti-fibrotic effects [64,65], as well as modulation of mitochondria function and energy metabolism [26,66].

Taken together, the finding that Bic reduced NAD^+^ level at 24 h (Figure 6a), inhibited SIRT1 and SIRT 3 (Figure 7a,b) with decreased ATP production rate in the RMC cells, as demonstrated recently [31], has apparently implicated the potential of Bic to induce renal damages.

### 3.9. PGC1α Was Upregulated by Bicalutamide Treatment

**Figure 8:**
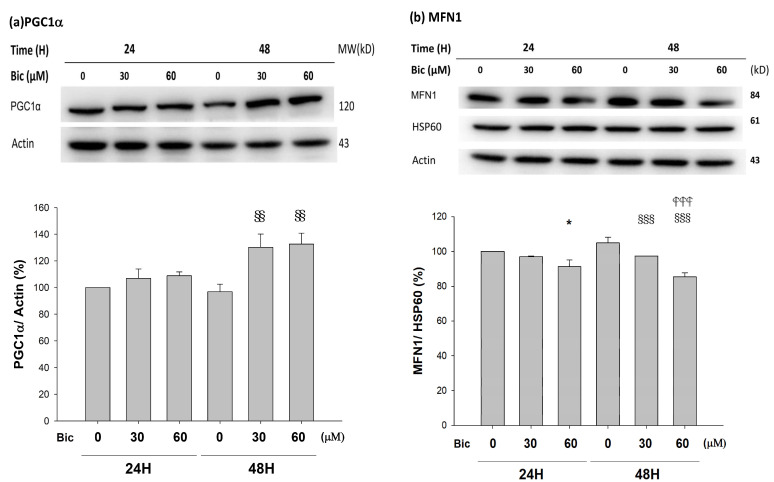

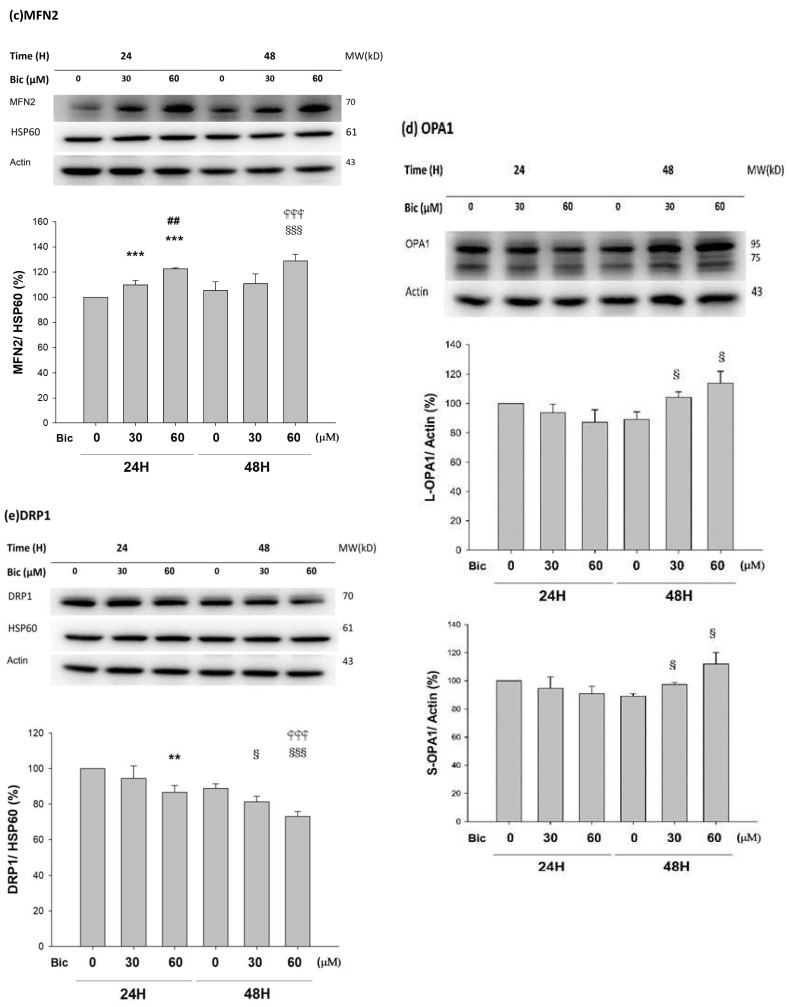
Representative western blot and densitometric analysis showing level of PGC1α (**a**), MFN1 (**b**), MFN2 (**c**), OPA1 long form (L-OPA1, 95kD) and short form (S-OPA1, 75kD) (**d**), and DRP1 (**e**) proteins in RMC cells following 0, 30, and 60 μM of bicalutamide treatment for 24 and 48 h. β-Actin is as cytosolic normalization control; HSP60 is as mitochondrial normalization control. Bar graph represents the mean ± SD of triplicate experiments: * *p* < 0.05, ** *p* < 0.01 *** *p* < 0.001 compared to Bic 0 μM at 24 h. ^##^ *p* < 0.01 compared to Bic 30 μM at 24 h. ^§^ *p* < 0.05, ^§§^ *p*< 0.01, ^§§§^ *p* < 0.001 compared to Bic 0 μM at 48 h. 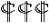 *p* < 0.001 compared to Bic 30 μM at 48 h.

PGC1α was upregulated at 48 h when treated with Bic (*p* < 0.05) (Figure 8a).

The PGC1α family is especially expressed in metabolically active tissues, such as the liver, kidneys, and brain, and controls global oxidative metabolism [22]. PGC1α family is responsible for two types of remodeling: (1) cellular remodeling through mitochondrial biogenesis [21,22,67], and (2) organelle remodeling through alteration in the intrinsic properties of mitochondria [67]. Mitochondrial biogenesis enhances metabolic pathways that ameliorate injury from aging, tissue hypoxia, glucose or fatty acid overload, and ROS, all of which contribute to the pathogenesis of acute and chronic kidney disease [68].

PGC1α is a pivotal determinant of renal recovery from injury by regulating NAD^+^ biosynthesis. PGC1α-dependent NAD^+^ biosynthesis links oxidative metabolism to renal protection [69]. PGC1α coordinately upregulated the enzymes that synthesize NAD^+^ de novo from amino acids, whereas PGC1α deficiency or AKI attenuated the de novo pathway [70]. PGC1α^−/−^ mice with renal ischemia developed local deficiency of the NAD^+^ precursor niacinamide, marked fat accumulation, and failure to re-establish normal function [69].

The dynamic modulation of PGC1α is affected by the energy metabolism acquired by proliferation and invasiveness of specialized cellular physiological condition in response to energy-demanding situations [22].

As seen, the severely reduced NAD^+^ after 24 h of Bic-treatment (Figure 6a) was followed by upregulation of PGC1α at 48 h (Figure 8a). Speculatively, such deficiency in NAD^+^ in complex I signaled PGC1α doing efforts to re-establish the NAD^+^ biosynthesis. However, according to Tran et al., due to local deficiency of the NAD^+^ precursor niacinamide [69], such upregulation of PGC1α to re-establish biogenesis eventually was in vain. Moreover, the import of transcriptionally upregulated PGC1α into the cytosol (Figure 8a) may be hindered by the inhibited TOMM20 (Figure 2).

The role of SIRT1 with PGC1α has been controversially cited. Gurd et al. evidenced that the deacetylation enzyme SIRT1 is not associated with oxidative capacity, with the notation that SIRT1 protein plays an obligatory regulatory role in the process of PGC1α-mediated mitochondrial biogenesis [71], and, meanwhile, SIRT1 was downregulated, while PGC1α was upregulated [71,72], which was consistent with our findings (Figure 7a and Figure 8a).

### 3.10. Mitochondria Dynamics Was Downregulated by Bicalutamide

Mitochondrial fusion is relevantly associated with several guanosine triphosphate hydrolases (GTPase), including mitofusins 1 and 2 (MFN1, MFN2), as well as optic atrophy 1 (OPA1). Both MFN1 and MFN2 are involved in outer-mitochondrial membrane (OMM) fusion, while OPA1 splicing is related with inner-mitochondrial membrane (IMM) fusion. In contrast, mitochondrial fission can be elicited by OMM-localizing fission 1 protein (FIS1) and the GTPase, dynamin-related protein (DRP1) [73]. Disruption in the mitochondria dynamics plays a role in the development of AKI and chronic kidney disease (CKD) [74].

As found, MFN1 was downregulated; in contrast, MFN2 was upregulated in a dose-dependent manner, at 48 h after treatment with Bic (Figure 8b,c). OPA1 upregulated at 48 h with Bic at 30 and 60 μM (Figure 8d), while DRP1 was downregulated in a time- and dose-dependent fashion after treatment with Bic at 30 and 60 μM, respectively (Figure 8e).

MFN1 and MFN2 have distinct roles in mitochondrial fusion [75]. To promote the formation or fusion of a branched network of elongated mitochondria by OPA1 required the outer membrane MFN1 but not MFN2 [75,76]. In contrast, literature revealed that MFN1 and MFN2 form homotypic and heterotypic complexes and act coordinately [77]. In order to rescue cell viability, the homotypic complexes are functional for fusion [77], which work together with s-OPA1 to stabilize the mitochondrial cristae and fusion [78]. The phenomenon that Bic upregulated OPA1 (Figure 8d), while simultaneously downregulating MFN1 (Figure 8b) and upregulating MFN2 (Figure 8c), implicates the rapid mitochondrial dynamic equilibrium between fusion and fission under the influence of Bic [79]. Thus, it is hard to tell, under the influence of Bic, a moderate inhibitor of ETC, which direction could be leading. Further experiments with higher doses and longer tracking time associated with more frequent checking points might uncover the true dynamics. Physiologically, the balance of fission and fusion of mitochondria can be tipped in either direction by changing a variety of factors, including the nutrient availability, the metabolic demands [80], and, speculatively, some medicines, such as Bic.

Genome-wide transcriptomic profiling revealed that the inhibition of fusion is associated with impaired OXPHOS, mtDNA depletion, and ROS production [81]. Mfn-null cells were shown a heterogeneity of mitochondria membrane potential and decrease of cellular growth and respiration [82,83]. Knockdown of the fusion regulator gene, OPA1, also inhibited the fusion process and resulted in a similar deficit [83]. While mitochondria deficient in DRP1 are morphologically bigger and functionally abnormal, the lack of mitochondria fission was also suggested to induce defective assembly of ETC complexes [74,84]. Suppression of either fusion or fission may result in reduction of mitochondria respiration and ATP production [74].

To summarize, the frequently regulated equilibrium between the continual cycles of mitochondrial fusion and fission is essential to maintain integrity of the organelle [73,85]. The mitochondria dynamic-related proteins were also modulated to respond to Bic-induced damage in the RMC cells.

## 4. Conclusions

Bicalutamide (Bic) is frequently used in androgen deprivation therapy for treating prostate cancer. However, ADT-induced hypogonadism exhibits the potential of leading to AKI. According to U.S. Food and Drug Administration, up to 37.5% of patients who have taken Bic therapy for 1–6 months may experience kidney failure.

**Figure 9:**
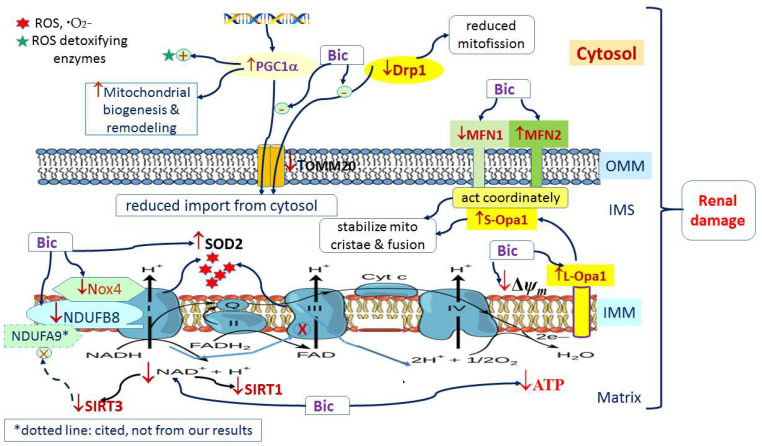
Graphic summary of mitochondria dynamics-related signaling pathways affected by bicalutamide.

Previously, we reported that Bic caused mitochondrial dysfunction via multiple mechanism of actions [31,32]. In this study, we have delineated a whole scope illustration indicating the actual mechanism how Bic affected the mitochondrial dynamics (Figure 9). Conclusively, damage to mitochondria is now understood to play a role in the pathogenesis of a wide range of disorders associated with hepatic, cardiac, and renal damages, that, in the past, seemingly had been considered unrelated. Long-term administration of Bic tends to cause a variety of adverse effects. The most rational approach is to understand the mechanisms underlying mitochondrial damage for specific medications, including Bic, and, on the other hand, to search for promising preventive or protective nutraceuticals to alleviate such detrimental effects.

## Supplementary Materials

The following supporting information can be downloaded at: https://www.mdpi.com/article/10.3390/jcm11010135/s1, Figure S1: MTT assay of RMC cells affected by bicalutamide in 25 mM hyperglycemic medium.
