# Correction: Chen et al. Bicalutamide Exhibits Potential to Damage Kidney via Destroying Complex I and Affecting Mitochondrial Dynamics. *J. Clin. Med.* 2022, *11*, 135

**DOI:** 10.3390/jcm15062381

**Published:** 2026-03-20

**Authors:** Kuan-Chou Chen, Chang-Rong Chen, Chang-Yu Chen, Chiung-Chi Peng, Robert Y. Peng

**Affiliations:** 1 Graduate Institute of Clinical Medicine, School of Medicine, College of Medicine, Taipei Medical University, 250 Wu-Xing St., Sin-Yi District, Taipei 11031, Taiwan; kuanchou@tmu.edu.tw; 2 Department of Urology, Taipei Medical University Shuang-Ho Hospital, New Taipei City 23561, Taiwan; 3 TMU-Research Center of Urology and Kidney, Taipei Medical University, Taipei 11031, Taiwan; 4 International Medical Doctor Program, Vita-Salute San Raffaele University, 20132 Milan, Italy; cherylcherylchen@gmail.com; 5 Program of Biomedical Sciences, College of Arts and Sciences, California Baptist University, Riverside, CA 92504, USA; eugenechen0529@gmail.com; 6 Department of Biotechnology, College of Medical and Health Care, Hungkuang University, No. 1018, Sec. 6, Taiwan Boulevard, Shalu District, Taichung 43302, Taiwan; robertpeng120@gmail.com

In the original publication [1], reference [29] is an online, third party advertisement. It has been deleted. The quoting sentence in the Introduction Section “A report from the U.S. Food and Drug Administration demonstrated that up to 37.5% of patients who have taken Bic therapy for 1–6 months may experience kidney failure [29]” has been removed. With this correction, the order of some references has been adjusted accordingly.

The authors state that the scientific conclusions are unaffected. This correction was approved by the Academic Editor. The original publication has also been updated.
