# Supplementary figures and images for: Bicalutamide Exhibits Potential to Damage Kidney via Destroying Complex I and Affecting Mitochondrial Dynamics
Source: J Clin Med. 2021 Dec 27;11(1):135. doi: 10.3390/jcm11010135 (PMC8745250; doi:10.3390/jcm11010135)

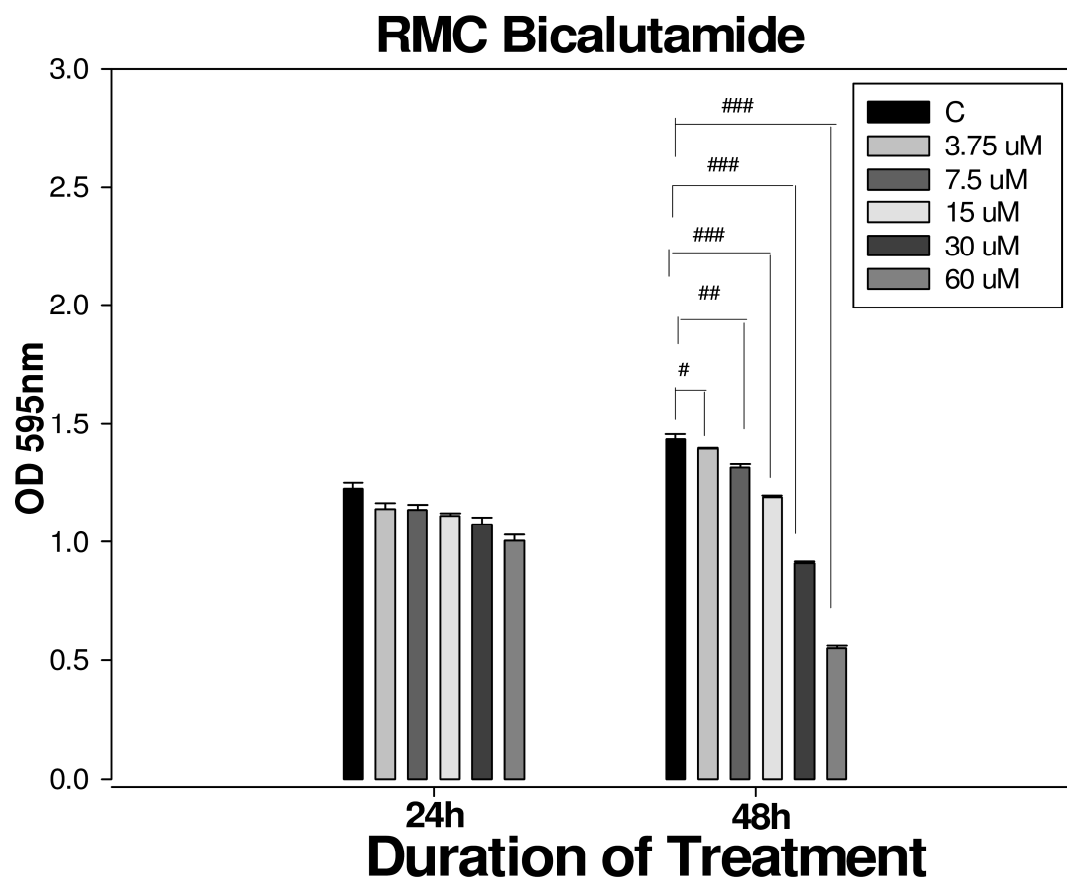

Figure S1. MTT assay of RMC cells affected by bicalutamide in 25 mM hyperglycemic medium.

Supplement: Supplementary file 1 [file jcm-11-00135-s001.zip › jcm-1442917-supplementary.pdf]
